# YY1 Regulates Melanocyte Development and Function by Cooperating with MITF

**DOI:** 10.1371/journal.pgen.1002688

**Published:** 2012-05-03

**Authors:** Juying Li, Jun S. Song, Robert J. A. Bell, Thanh-Nga T. Tran, Rizwan Haq, Huifei Liu, Kevin T. Love, Robert Langer, Daniel G. Anderson, Lionel Larue, David E. Fisher

**Affiliations:** 1 Department of Dermatology, Cutaneous Biology Research Center, Massachusetts General Hospital, Harvard Medical School, Boston, Massachusetts, United States of America; 2 Institute for Human Genetics, University of California San Francisco, San Francisco, California, United States of America; 3 Department of Epidemiology and Biostatistics, Department of Bioengineering and Therapeutic Sciences, The Eli and Edythe Broad Center of Regeneration Medicine and Stem Cell Research, University of California San Francisco, San Francisco, California, United States of America; 4 Division of Medical Oncology, Massachusetts General Hospital, Boston, Massachusetts, United States of America; 5 Children's Hospital Boston, Harvard Medical School, Boston, Massachusetts, United States of America; 6 Department of Chemical Engineering, Massachusetts Institute of Technology, Cambridge, Massachusetts, United States of America; 7 David H. Koch Institute for Integrative Cancer Research, Massachusetts Institute of Technology, Cambridge, Massachusetts, United States of America; 8 Harvard–MIT Division of Health Sciences and Technology, Cambridge, Massachusetts, United States of America; 9 Institut Curie, Developmental Genetics of Melanocytes, U1021 INSERM, UMR 3347 CNRS, Orsay, France; Yale School of Medicine, United States of America

## Abstract

Studies of coat color mutants have greatly contributed to the discovery of genes that regulate melanocyte development and function. Here, we generated *Yy1* conditional knockout mice in the melanocyte-lineage and observed profound melanocyte deficiency and premature gray hair, similar to the loss of melanocytes in human piebaldism and Waardenburg syndrome. Although YY1 is a ubiquitous transcription factor, YY1 interacts with M-MITF, the Waardenburg Syndrome IIA gene and a master transcriptional regulator of melanocytes. YY1 cooperates with M-MITF in regulating the expression of piebaldism gene *KIT* and multiple additional pigmentation genes. Moreover, ChIP–seq identified genome-wide YY1 targets in the melanocyte lineage. These studies mechanistically link genes implicated in human conditions of melanocyte deficiency and reveal how a ubiquitous factor (YY1) gains lineage-specific functions by co-regulating gene expression with a lineage-restricted factor (M-MITF)—a general mechanism which may confer tissue-specific gene expression in multiple lineages.

## Author Summary

Skin and hair pigmentation is among the most identifiable human traits. Disorders of pigment cells, melanocytes, result in multiple hypopigmentation conditions. Here, we described the phenotype of loss of a ubiquitous transcription factor YY1 in mouse melanocytes, which is reminiscent of certain human hypopigmentation conditions. We revealed at a molecular level that YY1 cooperates with a melanocyte-specific transcription factor M-MITF to regulate survival and pigmentation gene expression. This study is the first report of YY1 function in melanocyte lineage, and it reveals how a ubiquitous transcription factor gains lineage-specific functions by co-regulating gene expression with a lineage-restricted transcription factor.

## Introduction

Waardenburg syndrome, Tietz syndrome and piebaldism represent disorders of melanocyte migration, proliferation, or survival during embryonic development and are characterized by stable congenital white patches of the skin and hair. They are caused by mutations in various genes, including *PAX3* (paired-box 3), *SOX10* (sex-determining region Y-box 10), *MITF* (microphthalmia-associated transcription factor), *EDN3* (endothelin 3), *EDNRB* (endothelin receptor B) and *KIT*, resulting in hypopigmentation due to a lack of melanocytes rather than a lack of pigment in viable melanocytes, as occurs in albinism [1], [2], [3]. Among these genes, MITF is one of the earliest melanocyte-specific transcription factors and is a master regulator of melanocyte development and function. In humans, germline loss-of-function mutations of *MITF* are associated with Waardenburg Syndrome (WS) type IIA and Tietz syndrome, autosomal dominant conditions which exhibit melanocytic deficiencies and pigmentation abnormalities together with variable severity of sensorineural deafness [4], [5], [6]. M-MITF is the melanocyte-specific isoform of MITF. The importance of MITF in melanocyte differentiation is highlighted by its direct and lineage-specific transcription of essential pigmentation enzymes and melanosome components, e.g., *tyrosinase* (*TYR*), *dopachrome tautomerase* (*DCT*) and *silver* (*SILV*). MITF also regulates the receptor tyrosine kinase KIT [7], which is necessary for the survival and dispersal of melanocyte precursors from the migration staging area. Inactivating mutations or deletion of *KIT* lead to piebaldism in humans, with loss of melanocytes typically restricted to the hair and skin [3], [8], [9]. MITF thus has diverse functions in melanocyte differentiation, growth and survival pathways [10], [11], [12].

Yin Yang 1 (YY1) is a ubiquitously expressed zinc-finger transcription factor. It can act as transcriptional repressor or activator [13]. The essential role of YY1 in development is underscored by the fact that genetic ablation of *Yy1* in mice resulted in peri-implantation lethality [14]. During B cell and oligodendrocyte lineage development, YY1 functions as a pro-differentiation factor [15], [16]. In mouse spermatogenesis, YY1 is required for maintaining heterochromatin structure integrity [17]. YY1 thus has important functions in several lineages, but given its ubiquitous expression in the majority of tissues, it is not known whether it is able to regulate select genes in a lineage-specific manner. This paper reports a key role for YY1 in melanocytic lineage development and describes how melanocyte-specific functions of YY1 may be directed by its interaction with M-MITF.

## Results

### YY1 is required for melanocyte development and survival in vivo and in vitro

**Figure 1:**
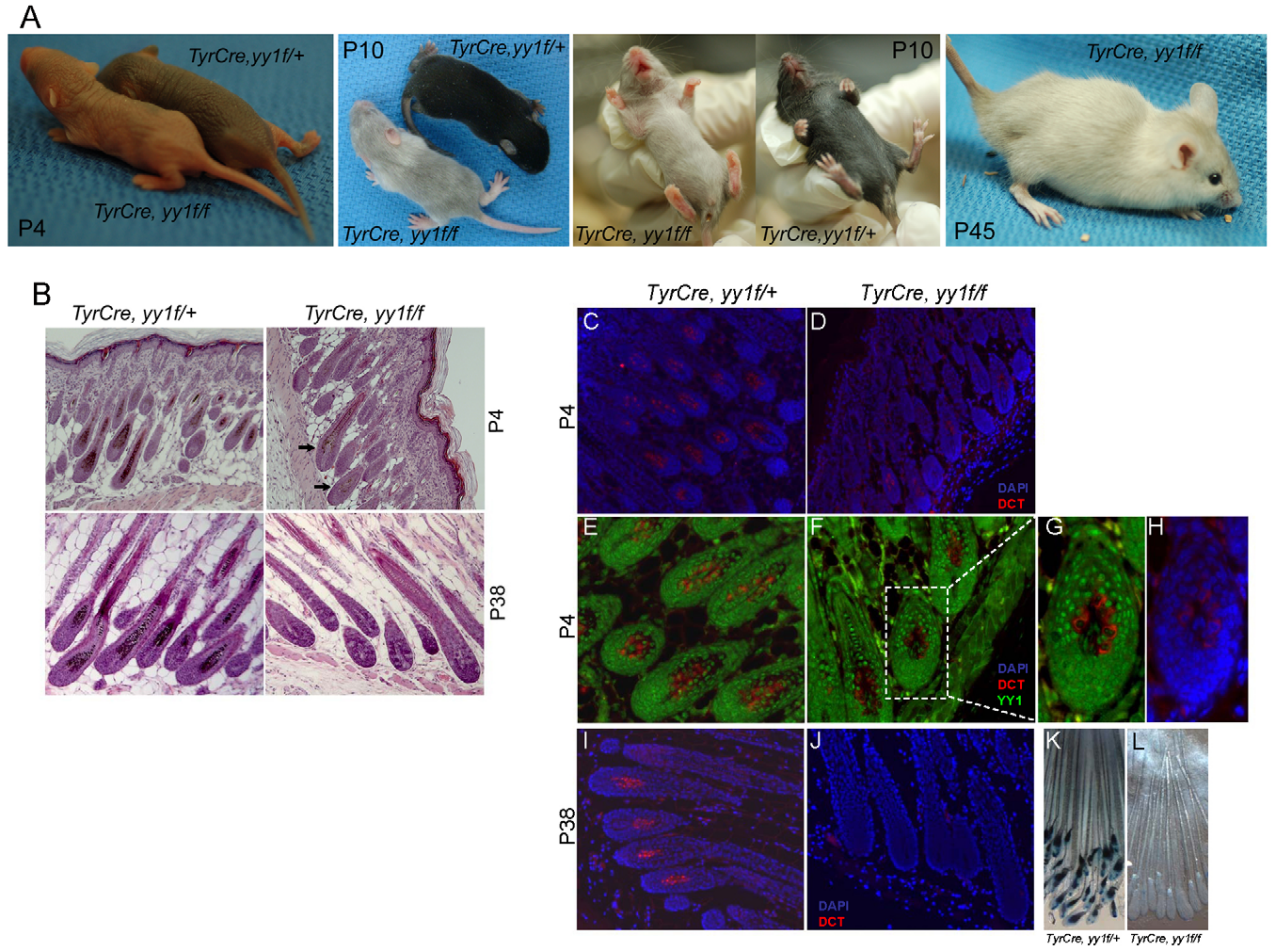
YY1 is required for melanocyte development in vivo. (A) Skin and hair pigmentation phenotype of *TyrCre, yy1f/fl* in the first (P4, P10) and second (P45) hair cycles. (B) H&E staining of hair follicles from skin sections of *TyrCre, yy1f/+* and *TyrCre, yy1f/f* mice in the first (P4) and second (P38) hair cycles. Arrows point to the hair follicles still containing pigment. (C,D) Immunofluorescence staining of DCT (red) in P4 hair follicles of *TyrCre, yy1f/+* (C) and *TyrCre, yy1f/f* (D) mice. Skin sections were stained with goat anti-DCT primary antibody and donkey anti-goat Alexa 594 secondary antibody. Nuclei were counterstained with DAPI (blue). (E,F) Immunofluorescence staining of DCT (red) and YY1 (green) in P4 hair follicles of *TyrCre, yy1f/+* (E) and *TyrCre, yy1f/f* (F) mice. Skin sections were double-stained with goat anti-DCT and rabbit anti-YY1 primary antibodies and then donkey anti-goat Alexa 594 and goat anti-rabbit Alexa 488 secondary antibodies. (G,H) Zoom-in view of the dashed box area in (F). DAPI stain is in blue in (H). (I,J) Immunofluorescence staining of DCT (red) in P38 hair follicles of *TyrCre, yy1f/+* (I) and *TyrCre, yy1f/f* (J) mice. Nuclei were stained with DAPI (blue). (K&L) XGal stain of whole-mount skin sections from P38 *TyrCre, yy1f/+, Dct-LacZ* (K) and *TyrCre, yy1f/f, Dct-LacZ* (L) mice.

To study the function of YY1 in melanocyte development, we generated melanocyte-specific *Yy1* conditional knockout mice (*TyrCre, yy1f/f*) by crossing *yy1flox/flox* mice [18] with TyrCre mice in which Cre expression is constitutively driven by the melanocyte-specific tyrosinase (Tyr) promoter [19]. Cre-mediated genetic recombination starts from embryonic day 10.5. *TyrCre, yy1f/f* mice were born at the expected Mendelian ratio. Shortly after birth (P4), *TyrCre, yy1f/f* mice showed profoundly lighter skin pigmentation compared with littermate controls *TyrCre, yy1f/+* (Figure 1A, P4). In the first hair cycle (P0–P28), ventral hairs of *TyrCre, yy1f/f* mice were essentially devoid of pigment (Figure 1A, P10). Hairs from dorsal skin of *TyrCre, yy1f/f* mice were much less pigmented than those in control mice (Figure 1A, P10), and H&E sections of dorsal skin revealed small amounts of residual hair follicle melanin (Figure 1B, P4, arrows). As indicated by immunofluorescence (Figure 1C–1H), the residual dorsal melanocytes (DCT positive, Figure 1D) continued to express YY1 (compare nuclear YY1 signal to cytoplasmic DCT, Figure 1G), indicating incomplete Cre-mediated deletion. MITF expression was not affected in the residual hair follicle melanocytes of P4 *TyrCre, yy1f/f* mice (Figure S1A). In the second hair cycle anagen phase (P28–P42), new dorsal hair follicles of *TyrCre, yy1f/f* mice completely lacked melanin pigment (Figure 1B, P38) as well as DCT positive melanocytes (Figure 1I and 1J) and corresponded to subsequent white dorsal fur (Figure 1A, P45), indicating an ongoing need for YY1 in post-developmental melanocytes. Further support for melanocyte absence, rather than absence of pigment within viable melanocytes, came from *TyrCre, yy1f/f, Dct-lacZ* mice, which carry a *lacZ* reporter under the control of the melanocytic-specific *Dct* promoter [20]. XGal staining of whole-mount skin sections confirmed the absence of melanocytes or pigment in hair follicles of *TyrCre, yy1f/f, Dct-lacZ* mice at the anagen phase of the second hair cycle (P38) (Figure 1K and 1L, Figure S1B). Collectively these data suggest that YY1 is required for melanocyte development and post-developmental survival in vivo.

**Figure 2:**
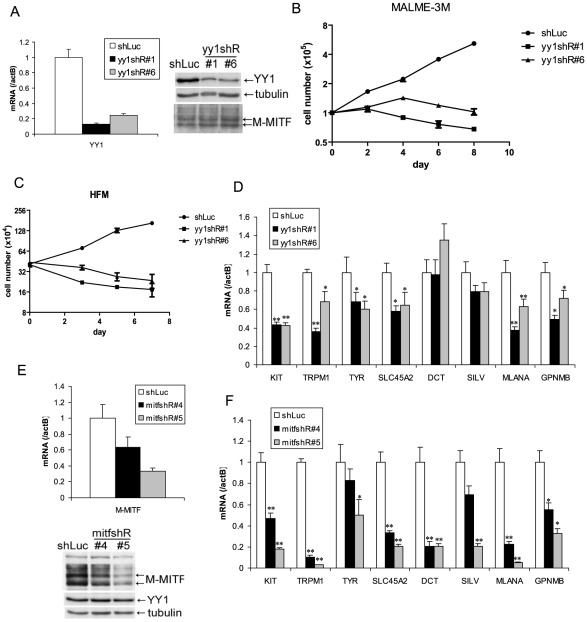
YY1 is required for melanocyte survival and the expression of melanocyte differentiation markers in vitro. (A,E) Knockdown of endogenous YY1 (A) and MITF (E) in MALME-3M cells. MALME-3M cells were infected with lentivirus carrying YY1 shRNA (yy1shR#1 & #6), MITF shRNA (mitfshR#4 & #5) or control shRNA (shLuc). After overnight infection, cells were selected with puromycin for 3 days. Total RNA and cell lysates were harvested for mRNA and protein measurements by RT-qPCR and western blotting. mRNA levels were normalized to β-actin (actB). (B) Growth curve of YY1 knockdown MALME-3M cells. After 3 days of puromycin selection of YY1 knockdown and control cells as in (A), cells were re-seeded at 1×10^5^ per 6 cm plate. Cell numbers were counted every other day. (C) Growth curve of YY1 knockdown HFM cells. HFM were infected and selected with puromycin as in (A). After puromycin selection, cell numbers were counted on days 0, 3, 5, 7. (D,F) mRNA levels were quantitated for *KIT* and multiple melanocyte differentiation genes in YY1 (D) and MITF (F) knockdown cells. Error bars represent s.d. of triplicates. *, p<0.05; **, p<0.01 (Student t-test).

To determine whether YY1 is also required for melanocytic cell survival in vitro, we stably knocked down endogenous YY1 using two lentiviral shRNAs (yy1shR#1&#6) (Figure 2A). Prolonged depletion of YY1 led to decreased cell numbers in both YY1 knockdown MALME-3M melanoma cells and human foreskin primary melanocytes (HFM), similar to MITF knockdown (Figure 2B and 2C, Figure S2A). While a decrease in cell numbers clearly indicates cell death, it is plausible that there could also be anti-proliferative effects in some (surviving) cells, a possibility that will require additional study.

### YY1 is required for the expression of melanocyte survival and differentiation genes

Melanocyte deficiency and premature gray hair in melanocyte-specific *Yy1* conditional knockout mice are reminiscent of the melanocyte deficiency phenotype in human Waardenburg syndrome and related disorders and suggest that YY1 may transcriptionally regulate genes important for lineage development or survival. We therefore performed expression profiling analysis using MALME-3M melanoma cells with or without YY1 knockdown. Although the Waardenburg syndrome genes *PAX3*, *SOX10*, *MITF*, *EDN3* and *EDNRB* were not dramatically affected at the mRNA level (Table S1), expression of the piebaldism gene *KIT* was significantly down-regulated upon loss of YY1 (Table S1 and Figure 2D). It is currently unclear whether the diminished MALME-3M viability upon loss of YY1 is mediated by suppression of KIT expression, or the other survival factors. As demonstrated by immunofluorescence, KIT protein was significantly reduced in the residual hair follicle melanocytes (DCT positive) in P4 *TyrCre, yy1f/f* mice (Figure S2B). The mRNA levels of multiple melanocyte differentiation factors *TYR*, *SLC45A2*, *MLANA*, *TRPM1*, and *GPNMB*, were also down-regulated in YY1 knockdown cells (Table S1 and Figure 2D). These melanocyte differentiation genes are well-known direct targets of MITF [11]. Indeed, MITF knockdown also strongly down-regulated these and other pigmentation genes (e.g. *DCT* and *SILV*, whose MITF binding sites have been previously reported [21], [22]) (Figure 2E and 2F). *KIT* has been shown to be a target of MITF in mast cells [7]. MITF knockdown also reduced the expression of KIT in MALME-3M melanoma cells (Figure 2F). *BCL2*, another known target of MITF [23], was modestly down-regulated in both MITF and YY1 knockdown MALME-3M cells (Figure S2C).

As knockdown of YY1 did not change MITF protein levels (Figure 2A), the fact that it significantly affected the levels of many MITF target genes suggested that YY1 might functionally cooperate with MITF. To explore this possibility, we performed expression profiling analysis with MITF knockdown melanoma cells (Figure 2E). We found that 1241 RefSeq genes showed significantly reduced expression after MITF knockdown (Table S1). YY1's DNA binding sequence was one of the top five motifs showing the greatest enrichment in open chromatin regions of the MITF-responsive gene promoters (Materials and Methods, and Table S2), further strengthening the possibility that YY1 might cooperate with MITF.

### YY1 interacts with MITF

**Figure 3:**
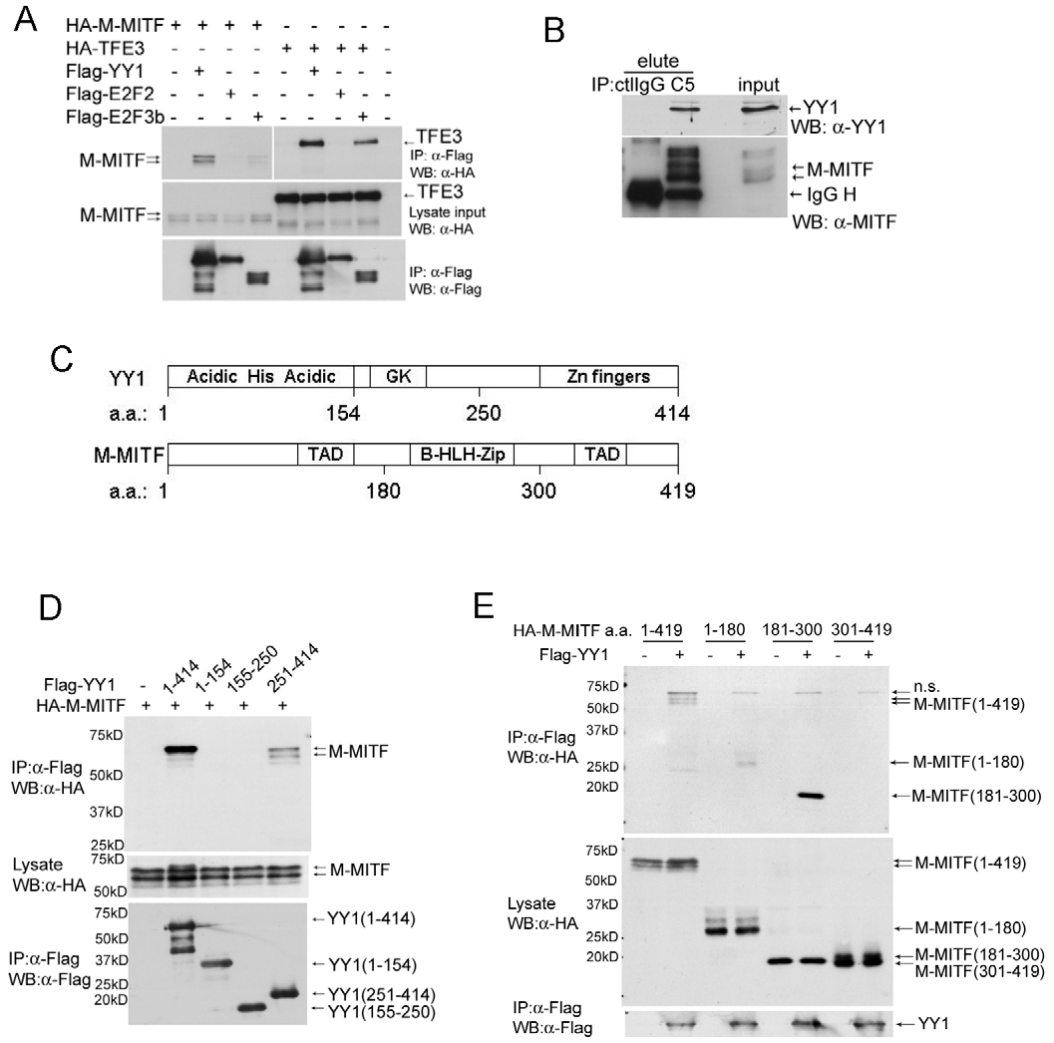
YY1 interacts with MITF. (A) Interaction between Flag-tagged YY1 and HA-tagged M-MITF in 293T cells. Total cell lysate of 293T cells transfected with indicated plasmids was immunoprecipitated with anti-Flag M2 agarose beads. Immunocomplex and lysate input were analyzed by western blotting with anti-Flag and anti-HA antibodies. (B) Endogenous protein interaction between YY1 and MITF. Total cell lysate of MALME-3M was immunoprecipitated with mouse control IgG (ctlIgG) or mouse anti-MITF monoclonal antibody (C5). Immunocomplex (elute) and lysate input were analyzed by western blotting with anti-MITF and anti-YY1 antibodies. (C) Schematic diagrams of YY1 and M-MITF proteins. GK, glycine and lysine rich region; TAD, transcription activation domain; b-HLH-Zip, basic helix-loop-helix leucine zipper. (D) M-MITF interacts with the C-terminal domain (a.a. 251–414) of YY1. (E) YY1 interacts with the N-terminal TAD (a.a. 1–180) and the central b-HLH-Zip (a.a. 181–300) of M-MITF.

To test whether YY1 and MITF physically interact in a protein complex, we first co-transfected expression constructs of Flag-tagged YY1 and HA-tagged M-MITF or a close MiT family member TFE3 into 293T cells. Flag-tagged YY1 co-immunoprecipitated with M-MITF as well as with TFE3 (Figure 3A). Interaction of TFE3 with E2F3b but not with E2F2 served as positive and negative controls respectively, as previously described [24]. Endogenous YY1 also co-immunoprecipitated with endogenous MITF in MALME-3M cells (Figure 3B). YY1 contains an N-terminal histidine-rich region (His) flanked by acidic amino acids (Acidic), a central glycine/lysine-rich region (GK) and C-terminal zinc fingers [25] (Figure 3C). MITF contains a central basic helix-loop-helix leucine-zipper domain (b-HLH-Zip) and two transcription activation domains (TAD) (Figure 3C and reference therein [10]). Expression constructs encoding full-length or truncated mutants of YY1 and M-MITF revealed required binding domains for YY1-MITF complex formation: M-MITF interacted with full-length as well as the C-terminal domain (amino acid 251–320) of YY1 (Figure 3D, Figure S3A); YY1 interacted with full-length, N-terminal TAD (amino acid 1–180) and central b-HLH-Zip (amino acid 181–300) of M-MITF, but not with C-terminal TAD (amino acid 301–419) (Figure 3E, Figure S3B). A smaller b-HLH-Zip domain (amino acid 181–264) of MITF showed a weaker interaction with YY1 compared with the full length central domain (Figure S3B), suggesting that the whole b-HLH-Zip domain is important in mediating the strongest interaction

### YY1 cooperates with MITF in gene transcription

**Figure 4:**
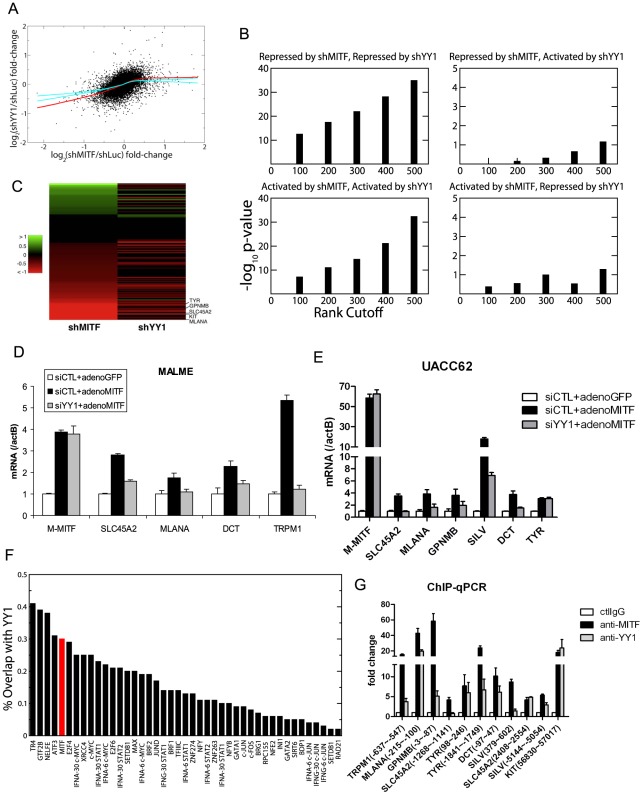
YY1 cooperates with MITF in gene transcription. (A) Scatter plot of mean log fold-changes in MALME-3M and UACC62 cells treated with MITF shRNA (shMITF) and YY1 shRNA (shYY1) relative to the control (shLuc). The red line is the average of 100 lowess regression curves fitted to 100 bootstrap simulations. The cyan curves represent the upper and lower bounds of 1000 lowess curves fitted to the data obtained by randomizing the x-coordinates of differentially expressed genes after YY1-shRNA. The fact that the observed fit lies well outside the error bounds of randomized data in the lower left quadrant shows that YY1 and MITF co-activate a statistically significant number of common target genes. (B) Fisher's exact test p-values are plotted for the significance of the overlap between the top ranking differentially expressed genes after shMITF and shYY1, where the differential expression was ranked by RSA analysis p-values. Statistical significance is observed for genes that are either co-repressed or co-activated by MITF and YY1, but not for genes on which MITF and YY1 have antagonistic effects. (C) Gene Ontology analysis found 131 genes to play a role in melanosome function and pigmentation regulation. The heatmap shows the log expression fold-change of those 131 genes after MITF and YY1 knockdown. (D,E) Loss of YY1 inhibits M-MITF-dependent transcriptional up-regulation of multiple melanocytic markers. MALME-3M cells (D) or UACC62 cells (E) were transfected with 10 nM of YY1 siRNA (siYY1) or control siRNA (siCTL). After 24 h, cells were infected with adenovirus encoding cDNA of M-MITF or GFP at MOI 500 (D) or 100 (E). Cells were harvested 48 hours post infection. mRNA levels of the different genes were measured by RT-qPCR and normalized to β-actin (actB). Error bars represent s.d. of triplicates. (F) The fraction of transcription factor (TF) binding sites overlapping with YY1 was computed for 42 TFs mapped by the ENCODE consortium in the K562 cell line (black bar). The overlap between MITF binding sites in 501MEL and YY1 binding sites in MALME-3M (red bar) was 30%. (G) Co-localization of YY1 and MITF at the proximal promoter of multiple melanocyte differentiation markers in MALME-3M cells. ChIP-qPCR primer position relative to the transcription start site is indicated in brackets. Data are normalized to control IgG ChIP. Error bar represents s.d. of triplicates.

Using the expression profiling data, we further analyzed the cooperation between YY1 and MITF. Figure 4A shows the scatter plot of log fold-change of gene expression in MITF and YY1 knockdown cells relative to that in control cells. The bagged regression curve (red line) shows a significant association between the sets of genes co-activated by MITF and YY1, as well as genes co-repressed by MITF and YY1. We also compared the overlap between the top ranking differentially expressed genes after MITF knockdown or YY1 knockdown. Statistical significance is observed for genes that are either co-repressed or co-activated by MITF and YY1, but not for genes on which MITF and YY1 have antagonistic effects (Figure 4B). The statistical significance is seen to be robust and independent of the number of top ranking differentially expressed genes (Figure 4B). Of note, MITF and YY1 knockdown effects correlated for 131 pigmentation-related genes (Figure 4C).

Although knockdown of endogenous YY1 affected the basal transcription of multiple melanocyte differentiation genes (Figure 2D), overexpression of YY1 alone did not affect the expression of these genes (data not shown). In MALME-3M (high endogenous M-MITF level) and UACC62 cells (low endogenous M-MITF level), overexpression of M-MITF by adenovirus infection was sufficient to induce the transcription of multiple melanocyte differentiation genes (Figure 4D and 4E, Figure S4). However knockdown of YY1 inhibited the M-MITF-dependent transcriptional upregulation of most of these genes except *TYR*. These results suggest a dependency of M-MITF-induced transcription on YY1. Moreover this dependency is target specific: M-MITF depends on YY1 to upregulate the transcription of many target genes, like *SLC45A2*, *MLANA*, *GPNMB*, *SILV*, *DCT* and *TRPM1*; for other targets like *TYR*, although YY1 cooperates with MITF for its basal transcription (Figure 2D), knockdown of YY1 did not affect M-MITF-dependent transcriptional upregulation (Figure 4E), suggesting that M-MITF is a dominant factor in controlling their induction. The mechanistic basis for these variable dependencies will require further analysis.

To globally identify YY1 target genes in the melanocytic lineage, we performed a ChIP-seq analysis in MALME-3M melanoma cells. We obtained 15,940 peaks by using the Skellam statistic at a *p*-value cutoff of 10^−6^ (Materials and Methods, and Table S3). The overlap between recently published MITF binding sites [12] and our YY1 binding sites is 30% (Figure 4F), higher than the overlap for c-Myc, which has been previously documented to interact with YY1 [26]. Figure 4F shows that apart from some general transcription factors, MITF is the top differentiation factor whose binding sites significantly overlap with those of YY1. Gene ontology analysis found that MITF and YY1 co-regulate pathways involving mitochondria biogenesis, cytoskeleton, mitosis, as well as pigment granule and melanosome synthesis (Table S4).

We designed ChIP-qPCR primers at multiple reported MITF binding sites of the pigmentation genes [11], [12], and confirmed that YY1 co-localized with MITF on the proximal promoter of *TRPM1*, *MLANA*, *GPNMB*, *TYR* and *DCT* (Figure 4G). For *SLC45A2* and *SILV*, YY1 co-localized with MITF at the 2^nd^ nearest MITF binding sites [12] (Figure 4G). We obtained 8,899 melanocyte-specific YY1 binding sites by removing the sites that are found within 5 kb of YY1 ChIP-seq sites in GM12878, K562, and NT2D cell lines profiled by the ENCODE Consortium. We found that the nearest YY1 binding site on the *KIT* gene is localized in the 7th intron (peak at 56899 bp from the transcription start site). This YY1 binding site is melanocyte-specific and has an MITF binding site [12] within 50 bp, as confirmed by ChIP-qPCR (Figure 4G).

To validate the cooperativity of MITF and YY1 on a target gene promoter, we utilized a 700 bp upstream promoter region of TRPM1 fused to a firefly luciferase reporter [27]. There are three MITF consensus binding E-box sequences (E1, catgtg; E2, catgtg; E3, cacatg) and one YY1 consensus binding sequence (Y1, gccatc) within this promoter region, as shown in Figure S5. We mutated E-box sequences (mut2) or YY1 binding sequence (mut1) and found that mutation of the YY1 binding sequence modestly, but significantly reduced reporter activity by 22%, while mutation of MITF binding sequences dramatically reduced the reporter activity (Figure S5). While TRPM1 appears to be a melanocyte-specific gene (also called “melastatin” [27]), these experiments demonstrated a measurable contribution to its expression by the ubiquitous factor YY1.

## Discussion

In summary, our findings reveal a critical role of YY1 in melanocytic lineage development and function, as melanocyte-specific *Yy1* conditional knockout mice display complete loss of hair follicle melanocytes and premature gray hair after the first hair cycle. For a ubiquitous protein such as YY1 to gain cell type-specific functions, one of the most frequently adopted strategies is to interact with additional factors [25]. YY1 has been shown to directly interact with other ubiquitous factors, Smads, to activate cardiac development genes [28]. Here, we show that YY1 interacts with a lineage-specific transcription factor, M-MITF, to activate critical survival and pigmentation genes in melanocytic cells and that the transcriptional activity of M-MITF is dependent on YY1 for many target genes. Importantly, we demonstrate that despite its ubiquitous expression, YY1 acquires cell type-specific functions by interacting with a lineage-specific transcription factor. Since YY1 most strongly interacts with the intact b-HLH-Zip region of M-MITF (Figure 3E), which is present in other non-melanocytic isoforms of MITF, other isoforms of MITF might also interact with YY1 and potentially undergo functional effects within different lineages on the basis of similar biochemical forms of cooperativity. Novel mechanisms through which MITF and YY1 cooperate to confer survival to the melanocyte lineage are interesting subjects for future investigation.

## Materials and Methods

### Reagents

Antibodies used were anti-DCT (D-18), anti-YY1 (H-414) and anti-c-Kit (H-300) from Santa Cruz Biotechnology, anti-MITF (C5 mouse monoclonal and rabbit polyclonal) [22], anti-Flag (F3165, Sigma) and anti-HA (HA.11, Covance). Anti-Flag M2 agarose beads were obtained from Sigma-Aldrich.

### Generation and genotyping of TyrCre, yy1f/f mice

All animal work has been conducted according to MGH and national guidelines. Male *TyrCre* mouse (Cre transgene is on the X chromosome) [19] was crossed with *yy1f/f* female mouse to obtain *TyrCre*, *yy1f/+* female (F1). *TyrCre, yy1f/+* F1 female was then crossed with *yy1f/f* male to obtain *TyrCre*, *yy1f/f* male (F2). Genotyping primers are listed in Table S5.

### Histology and immunofluorescence staining

Mouse skin was fixed in 10% formalin after dissection and submitted to our histopathology core (Massachusetts General Hospital) for paraffin embedding, tissue section and H&E staining. For immunofluorescence staining, paraffin-embedded sections were washed twice in Xylene and then passed through 100%, 100%, 95% and 80% ethanol and H_2_O. Antigen retrieval was performed in EDTA buffer (5 mM Tris-HCl, 1 mM EDTA, PH 8.0) at 98°C for 20 min followed by cooling for 1 h. After PBS washing, tissue sections were blocked in 3%BSA/PBS for 1 h and then incubated with primary antibodies (1∶100 dilution) at 4°C overnight. After PBS washing three times, tissue sections were incubated with fluorescent secondary antibody (1∶1000 dilution) at room temperature for 1 h. Slides were mounted using VECTASHIELD mounting media with DAPI (Vector Laboratories). Fluorescent images were taken under Zeiss Axio Observer A1 microscope with AxioVision software.

### Whole-mount LacZ staining

Skin samples from *Dct-LacZ* mice were washed sequentially with PBS and LacZ wash buffer (2 mM MgCl_2_, 0.01% sodium deoxycholate, 0.02% Nonidet P40 in PBS PH 7.4). Samples were then incubated with LacZ solution (0.5 mg/ml XGal, 5 mM potassium ferricyanide, 5 mM potassium ferrocyanide in LacZ wash buffer) at 37°C for 24 h. After PBS washing, samples were fixed with fresh 4% paraformaldehyde/PBS for 10 min at room temperature. Stained (and fixed) samples were transferred onto microscope slides and mounted in Fluoromount-G mounting media (SouthernBiotech).

### Construction of YY1 and M-MITF expression plasmids

A plasmid containing cDNA encoding full-length human YY1 was obtained from the American Type Culture Collection (IMAGE clone ID, 5815774). The cDNA fragments encoding full-length or truncated YY1 were PCR amplified from IMAGE clone and inserted into p3XFLAG-CMV vector at EcoRI/BamHI sites. The cDNA fragments encoding truncated M-MITF were PCR amplified from pCDNA3-HA-hMi plasmid and inserted into pCDNA3-HA vector at EcoRI/NotI sites.

### MITF and YY1 RNAi

Lentiviral shRNA vector (pLKO.1) containing MITF and YY1 RNAi sequences were obtained from the Broad Institute RNAi Consortium (Cambridge, MA). Lentivirus was generated in 293T cells 72 h post transfection. Cells were infected with lentivirus for 24 h and selected in puromycin for 3 days. The shRNA sequences are: MITFshRNA#4, CGTGGACTATATCCGAAAGTT (sense); MITFshRNA#5, CGGGAAACTTGATTGATCTTT (sense); YY1shRNA#1, GCCTCTCCTTTGTATATTATT (sense) and YY1shRNA#6, GGGAGCAGAAGCAGGTGCAGAT (sense). Synthetic siRNA oligos targeting YY1 (siGENOME SMARTpool) and non-targeting control oligos were obtained from Thermo Fisher Scientific. Cells were transfected with siRNA using lipidoid reagent C12-113 and assayed 72 h post transfection.

### Lipidoid synthesis

Lipidoid delivery agent C12-113 was synthesized and characterized as previously described [29]. Briefly, 3 equivalents of 1,2-epoxydodecane was combined with 1 equivalent of 2,2′-diamino-N-methyldiethylamine (TCI America) in a glass scintillation vial. Reaction was stirred for 3 days at 90°C. Following synthesis, reaction mixture was characterized by MALDI-TOF mass spectroscopy to confirm mass of expected products. Synthesized material is used in *in vitro* biological assays without further purification. Lipidoid was dissolved in 25 mM NaOAc buffer (pH∼5.2) and added to solution of siRNA for complexation.

### Real-time quantitative PCR

The total volume of each reaction was 25 µl, including 12.5 µl 2× SYBR Green master mix (Bio-Rad), 0.25 µl reverse transcriptase (Qiagen), 1 µl of each primer (10 µM stock) and 100 ng of total RNA. Reverse transcription was carried out at 48°C for 30 min. Then 40 cycles of PCR reaction were carried out at 95°C for 15 s and 60°C for 30 s using 7500 Fast Real Time PCR system (Applied Biosystems). Data were acquired and analyzed with 7500 Fast System SDS software. qPCR primer sequences are listed in Table S5.

### Chromatin immunoprecipitation (ChIP)

Cells were fixed by adding formaldehyde to the culture media to a final concentration of 1% and incubated for 20 min at room temperature. Cells were harvested by scraping with ice cold PBS containing protease inhibitor (Roche). Cell pellets were re-suspended in SDS lysis buffer (1%SDS, 10 mM EDTA, 50 mM Tris-HCl, pH 8.0), incubated for 10 min on ice, and then sonicated to reduce DNA length to ∼500 base pairs. Samples were centrifuged to remove debris and then diluted 10 fold in IP buffer (0.01% SDS, 1.1% TritonX-100, 1.2 mM EDTA, 16.7 mM Tris-HCl, pH 8.0, 167 mM NaCl and protease inhibitors). To reduce nonspecific background, chromatin solution was pre-cleared with 80 µl of 50% protein A/G slurry containing 0.25 mg/ml sonicated salmon sperm DNA and 1 mg/ml BSA in TE (10 mM Tris, 1 mM EDTA, pH 8.0) for 1 h at 4°C. Antibodies were then added to pre-cleared chromatin solution and incubated overnight at 4°C. Protein A/G slurry (as used in the pre-clear step) were added to the sample and incubated for another 1 h at 4°C. The immuno-complexes were washed sequentially with buffer I (0.1% SDS, 1% TritonX-100, 2 mM EDTA, 20 mM Tris-HCl, pH 8.0, 150 mM NaCl), buffer II (0.1% SDS, 1% TritonX-100, 2 mM EDTA, 20 mM Tris-HCl, pH 8.0, 500 mM NaCl), buffer III (0.25M LiCl, 1% NP40, 1% sodium deoxycholate, 1 mM EDTA, 10 mM Tris-HCl, pH 8.0) and twice with TE. The immuno-complexes were eluted from beads with 1% SDS in 0.1M NaHCO_3_ twice for 15 min at room temperature. Crosslinks were reversed overnight at 65°C. Protein was digested with proteinase K for 1 h at 55°C. DNA was purified with QIAquick PCR purification kit (Qiagen). qPCR primers are listed in Table S5.

### Adenovirus infection

Adenoviruses encoding wild-type M-MITF or GFP were generated as previously described [27]. 2×10^5^ cells were plated in 6-well plates for 24 h. Cells were infected overnight with concentrated adenoviruses in complete media at MOI 500 for MALME-3M cells or MOI 100 for UACC62 cells and HFM. Media was changed the next day and total RNA was harvest with RNeasy plus mini kit (Qiagen) 48 h post infection.

### Expression profiling

mRNA expression in the melanoma cell lines MALME-3M and UACC62 were profiled using Affymetrix U133P2 microarrays. Each cell line had four samples, corresponding to two experiments treated with two independent shRNA constructs against MITF and two controls transfected with shRNA against luciferase. We also obtained two additional paired samples before and after siMITF (GSE16249, Gene Expression Omnibus). The data were normalized using RMA and the latest RefSeq probe mapping to the reference human genome [30], [31]. For each gene *g*, let *x_M1,g_* and *x_M2,g_* denote the fold-changes in shMITF-treated MALME-3M relative to the average control expression values in MALME-3M. Similarly denote the fold-changes in UACC62 and GSE16249 as *x_U1,g_*, *x_U2,g_* and *x_G1,g_*, *x_G2,g_*, respectively. In order to assess the significance of differential expression after knocking down MITF, we pooled together these 6 fold-changes for all genes and ranked them in an increasing order, and we then computed the minimum p-value for the rank distribution of each gene's fold-changes by using the hypergeometric test, as was done in the Redundant siRNA Activity (RSA) analysis [32]. The p-values were adjusted for multiple hypothesis testing and turned into q-values, as described in [33]. At a q-value cutoff of 10^−2^, 1241 RefSeq genes showed significantly reduced expression after MITF knockdown. A similar RSA analysis was performed for YY1 shRNA.

### Motif analysis

We found 814 open chromatin regions within the 1241 MITF-responsive RefSeq promoters in melanocytes, as recently mapped by the ENCODE Consortium. We also generated 814 matching random regions in the human genome. We then scanned the open chromatin and random regions with TRANSFAC and JASPAR position-specific scoring matrices (PSSM); the cutoff PSSM scores were chosen separately for each motif to minimize the binomial p-value for the over-representation or under-representation of each motif in the open chromatin regions of MITF-responsive promoters relative to random sequences.

### YY1 ChIP–seq

YY1 chromatin-immunoprecipitated DNA was sequenced by using Illumina Genome Analyzer II, yielding ∼15 million mappable reads. As a control, ∼9 million DNA fragments from randomly sonicated chromatin were also sequenced. Peaks were detected by using the Skellam distribution as a null model, similar to the Poisson null model used by MACS [34]. We used a p-value cutoff of 10^−6^; see Text S1 for further discussion. Regions containing satellites and rRNAs (ENCODE Duke Excluded Regions) were filtered out. Normalization of ChIP-seq data is described in detail in Text S1.

### Pathway analysis

We considered the genes down-regulated by both MITF and YY1 KD at a q-value cutoff of 0.01 and also bound by YY1 within 5 kb of transcriptional start site or in intron (Table S1). Gene ontology of those genes was analyzed by using DAVID (http://david.abcc.ncifcrf.gov).

### GSK cancer cell line expression analysis

954 CEL files were normalized together by using RMA [30] and the RefSeq CDF [31]. Replicate samples were averaged to yield summary expression levels for each cell line. Cell lines were then grouped into cancer subtypes, and the t-test was applied between each cancer subtype and the rest.

## Supporting Information

Figure S1 (A) Immunofluorescence staining of MITF in P4 hair follicles of *TyrCre, yy1f/+* and *TyrCre, yy1f/f* mice. Skin sections were stained with rabbit anti-MITF primary antibody and goat anti-rabbit Alexa 594 secondary antibody. MITF positive melanocytes were indicated by arrows. (B) Loss of melanoblasts and differentiated melanocytes in whisker hair follicles of P38 *TyrCre, yy1f/f, Dct-LacZ* mice. XGal stain of whole-mount whiskers from P38 *TyrCre, yy1f/+, Dct-LacZ* and *TyrCre, yy1f/f, Dct-LacZ* mice. The distribution of Dct-LacZ^+^ melanocyte stem cells (melanoblasts) is indicated by double arrow.
(PDF)

Figure S2 (A) Growth curve of MITF knockdown HFM cells. Experiment procedure is the same as in Figure 2C. (B) Immunofluorescence staining of KIT (green) and DCT (red) in P4 hair follicles of *TyrCre, yy1f/+* and *TyrCre, yy1f/f* mice. Skin sections were stained with rabbit anti-KIT and goat anti-DCT primary antibodies, followed by donkey anti-rabbit Alexa 488 and donkey anti-goat Alexa 594 secondary antibodies. (C) mRNA expression of BCL2 in MITF and YY1 knockdown MALME-3M cells. MITF and YY1 were knocked down in MALME-3M cells as in Figure 2. mRNA level of BCL2 was measured by RT-qPCR and normalized by beta-actin (actB).
(PDF)

Figure S3 Fine mapping of the interaction regions between M-MITF and YY1. Experiment procedure is the same as in Figure 3D and 3E.
(PDF)

Figure S4 mRNA levels of YY1 after siRNA knockdown. Experiment procedure is the same as in Figure 4D and 4E.
(PDF)

Figure S5 Mutation of MITF binding sites (E-boxes) or YY1 binding site on the TRPMI promoter inhibits luciferase reporter activity. 700 bp upstream promoter region of TRPM1 was cloned and fused with a firefly luciferase reporter (Miller AJ, 2004). There are three MITF consensus binding E-box sequences (E1, catgtg; E2, catgtg; E3, cacatg) and one YY1 consensus binding sequence (Y1, gccatc) within this promoter region. E1, E2 and E3 were mutated as in Miller AJ, 2004. Y1 site was mutated to gctgcc using QuickChange Site-Directed Mutagenesis kit (Stratagene). 0.2 µg of wild type (wt) or mutant (mut) firefly reporter constructed was co-transfected with 1 ng of renila construct into MALME-3M cells. Luciferase activity was measured by Dual-Luciferase reporter assay system (Promega). *p<0.05, ***p<0.001.
(PDF)

Table S1 Genes that are down-regulated after MITF and YY1 knock-down at RSA q-value cutoff of 1e-2 and also with a YY1 binding site either within 5 kb of transcription start site or in gene body.
(XLS)

Table S2 Enriched motifs in the promoters of genes that respond to MITF shRNA. The TF names show either TF.TRANSFAC_Matrix_ID or JASPAR matrix names.
(XLS)

Table S3 YY1 bindings locations in MALME-3M. The coordinates are in HG18.
(XLS)

Table S4 Gene ontology analysis of genes down-regulated by both MITF and YY1 knockdown at a q-value cutoff of 0.01 and also bound by YY1 within 5 kb of transcriptional start site or in intron.
(XLS)

Table S5 Genotyping and qPCR primer sequences.
(DOC)

Text S1 Supplemental methods.
(DOC)
