# Supplementary figures and images for: YY1 Regulates Melanocyte Development and Function by Cooperating with MITF
Source: PLoS Genet. 2012 May 3;8(5):e1002688. doi: 10.1371/journal.pgen.1002688 (PMC3342948; doi:10.1371/journal.pgen.1002688)

Figure S1

A

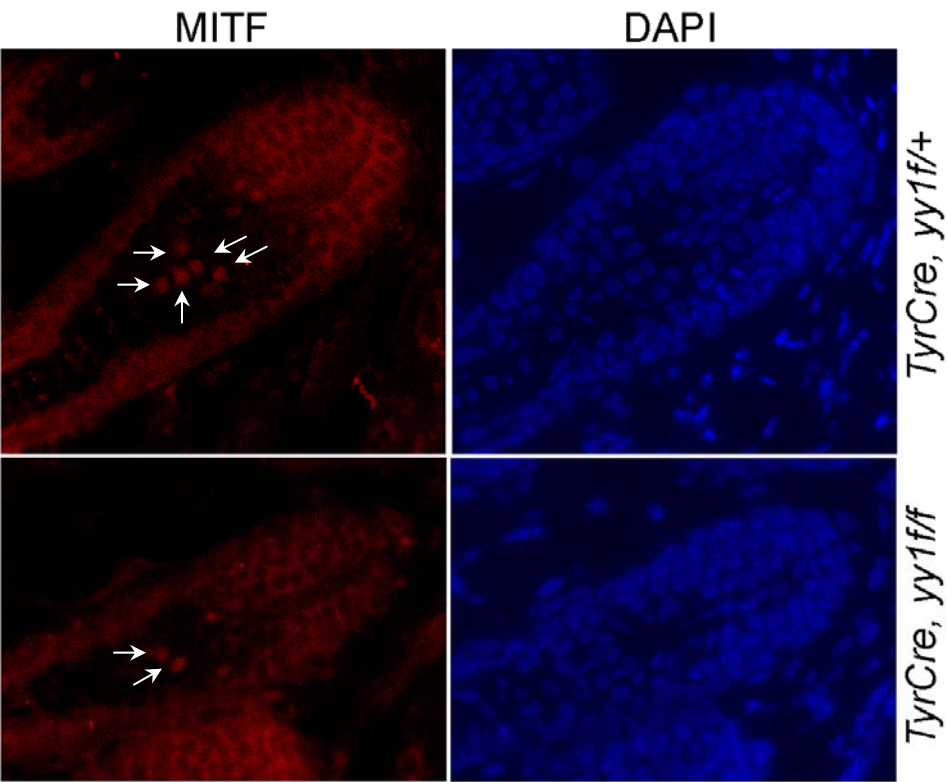

B

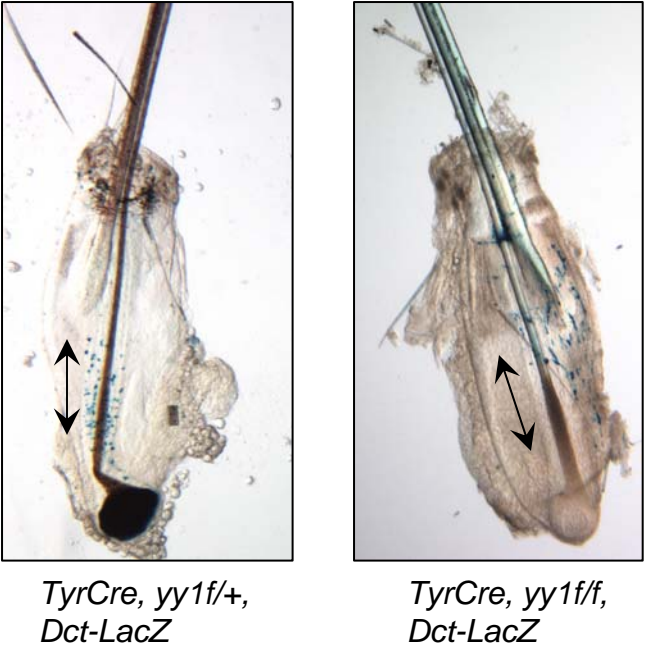

Supplement: Figure S1 — (A) Immunofluorescence staining of MITF in P4 hair follicles of TyrCre, yy1f/+ and TyrCre, yy1f/f mice. Skin sections were stained with rabbit anti-MITF primary antibody and goat anti-rabbit Alexa 594 secondary antibody. MITF positive melanocytes were indicated by arrows. (B) Loss of melanoblasts and differentiated melanocytes in whisker hair follicles of P38 TyrCre, yy1f/f, Dct-LacZ mice. XGal stain of whole-mount whiskers from P38 TyrCre, yy1f/+, Dct-LacZ and TyrCre, yy1f/f, Dct-LacZ mice. The distribution of Dct-LacZ+ melanocyte stem cells (melanoblasts) is indicated by double arrow. (PDF) [file pgen.1002688.s001.pdf]

Figure S2

A

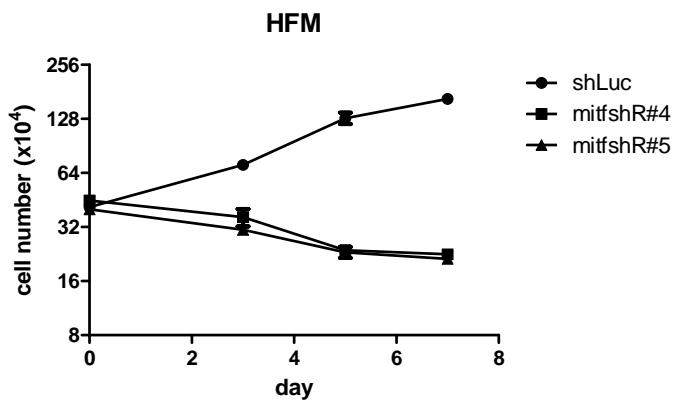

C

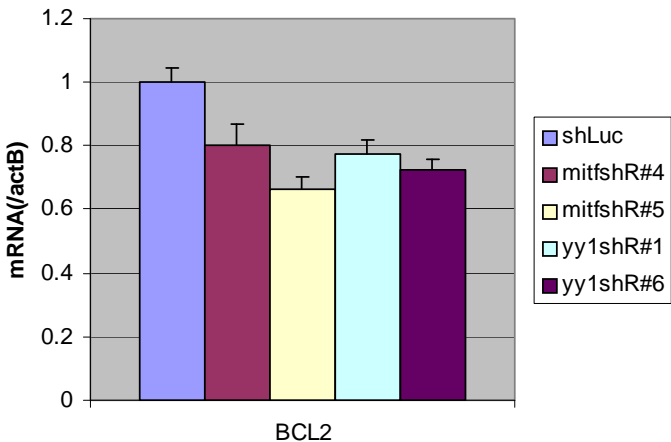

B

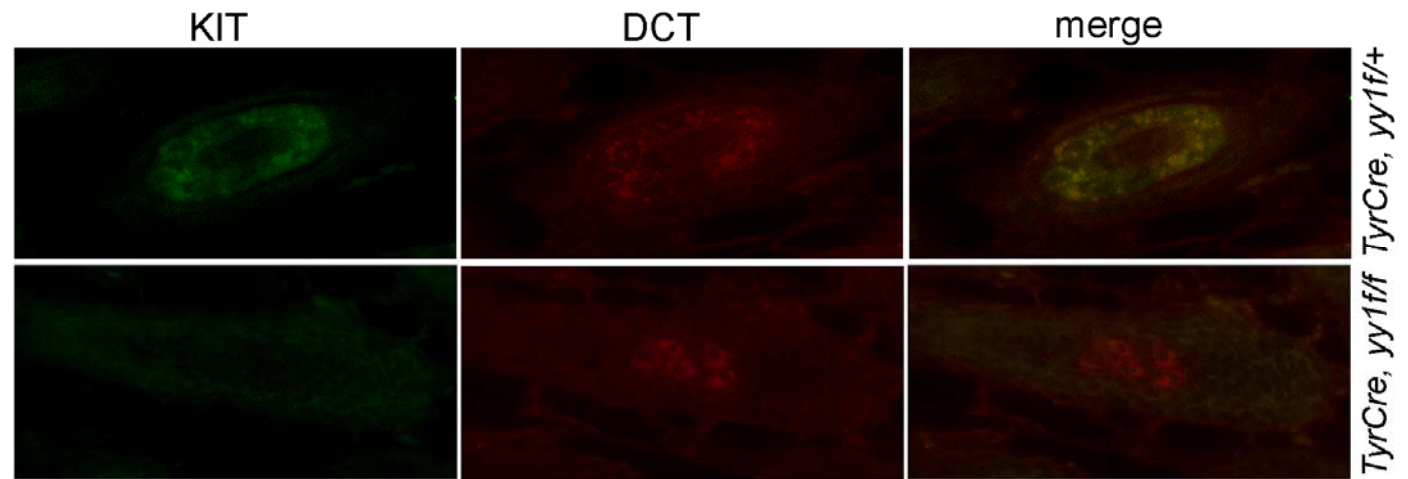

Supplement: Figure S2 — (A) Growth curve of MITF knockdown HFM cells. Experiment procedure is the same as in Figure 2C. (B) Immunofluorescence staining of KIT (green) and DCT (red) in P4 hair follicles of TyrCre, yy1f/+ and TyrCre, yy1f/f mice. Skin sections were stained with rabbit anti-KIT and goat anti-DCT primary antibodies, followed by donkey anti-rabbit Alexa 488 and donkey anti-goat Alexa 594 secondary antibodies. (C) mRNA expression of BCL2 in MITF and YY1 knockdown MALME-3M cells. MITF and YY1 were knocked down in MALME-3M cells as in Figure 2. mRNA level of BCL2 was measured by RT-qPCR and normalized by beta-actin (actB). (PDF) [file pgen.1002688.s002.pdf]

Figure S3

A

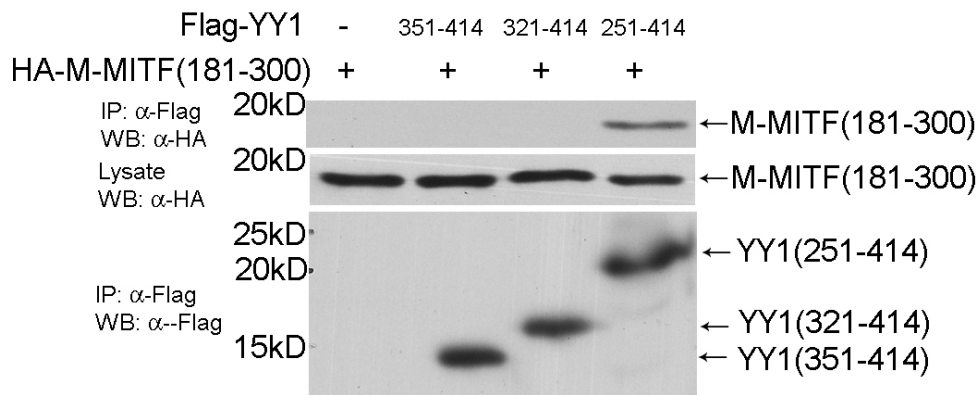

B

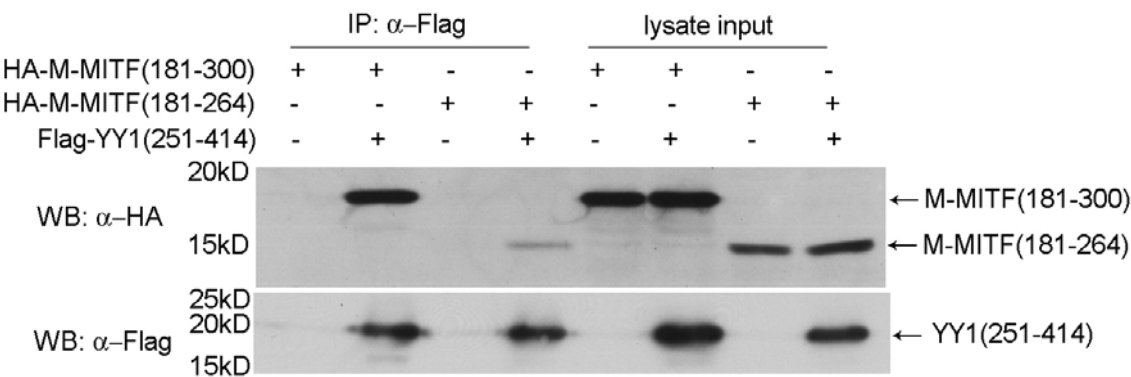

Supplement: Figure S3 — Fine mapping of the interaction regions between M-MITF and YY1. Experiment procedure is the same as in Figure 3D and 3E. (PDF) [file pgen.1002688.s003.pdf]

Figure S4

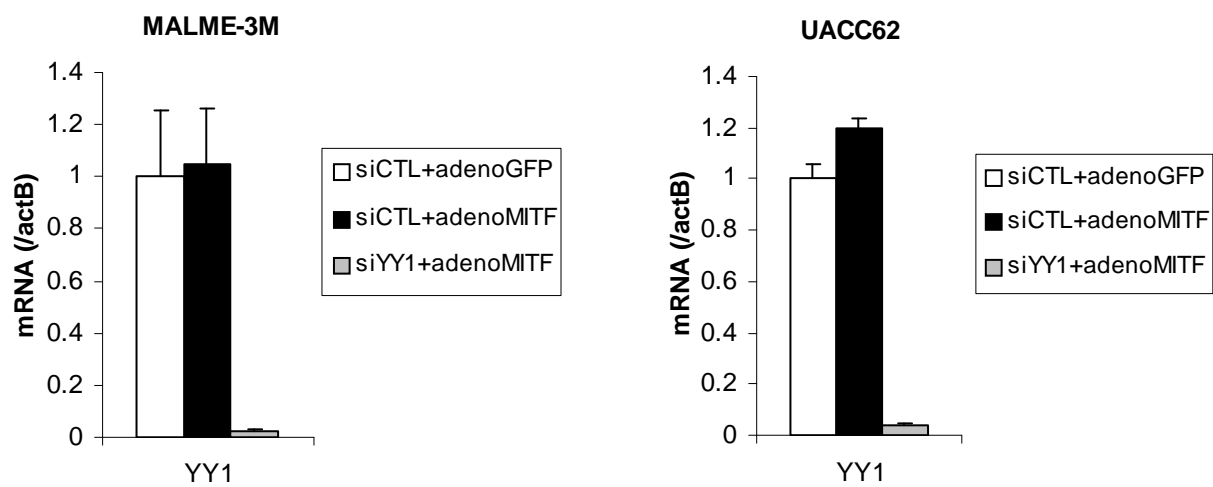

Supplement: Figure S4 — mRNA levels of YY1 after siRNA knockdown. Experiment procedure is the same as in Figure 4D and 4E. (PDF) [file pgen.1002688.s004.pdf]

Figure S5

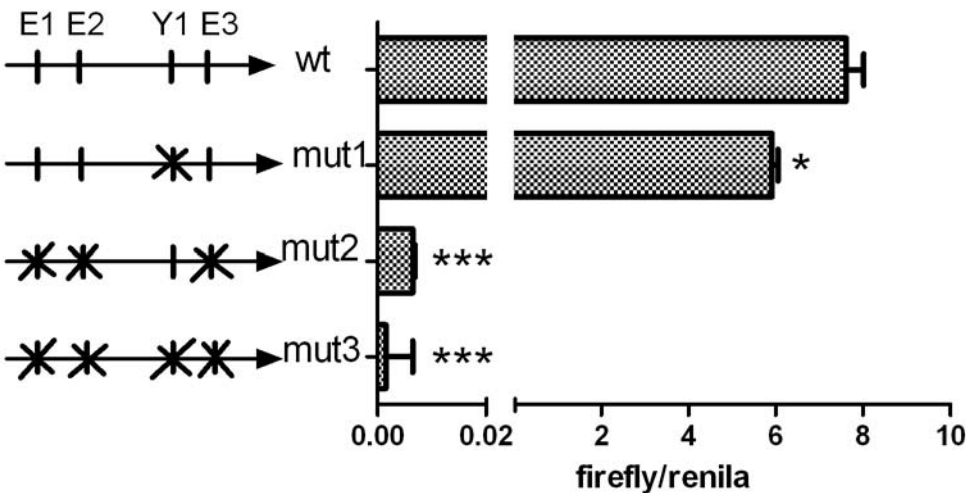

Supplement: Figure S5 — Mutation of MITF binding sites (E-boxes) or YY1 binding site on the TRPMI promoter inhibits luciferase reporter activity. 700 bp upstream promoter region of TRPM1 was cloned and fused with a firefly luciferase reporter (Miller AJ, 2004). There are three MITF consensus binding E-box sequences (E1, catgtg; E2, catgtg; E3, cacatg) and one YY1 consensus binding sequence (Y1, gccatc) within this promoter region. E1, E2 and E3 were mutated as in Miller AJ, 2004. Y1 site was mutated to gctgcc using QuickChange Site-Directed Mutagenesis kit (Stratagene). 0.2 µg of wild type (wt) or mutant (mut) firefly reporter constructed was co-transfected with 1 ng of renila construct into MALME-3M cells. Luciferase activity was measured by Dual-Luciferase reporter assay system (Promega). *p<0.05, ***p<0.001. (PDF) [file pgen.1002688.s005.pdf]
