# Supplementary material for: YY1 Regulates Melanocyte Development and Function by Cooperating with MITF
Source: PLoS Genet. 2012 May 3;8(5):e1002688. doi: 10.1371/journal.pgen.1002688 (PMC3342948; doi:10.1371/journal.pgen.1002688)
Supplement: Table S5 — Genotyping and qPCR primer sequences. (DOC) [file pgen.1002688.s010.doc]

Table S5. Primer information

Genotyping

| Gene | Forward | Reverse |
| --- | --- | --- |
| YY1 | ACCTGGTCTATCGAAAGGAAGCAC | GCTTCGCCTATTCCTCGCTCATAA |
| TyrCre | ATGGGGAAATTTTGGGGTAG | CAGCAGACACCAAGGAAACA |
| LacZ | GATCCAGCGATACAGCGCGTC | TTTAACGCCGTGCGCTGTTCG |

RT-qPCR

| Gene | Forward | Reverse |
| --- | --- | --- |
| MITF | CATTGTTATGCTGGAAATGCTAGAA | GGCTTGCTGTATGTGGTACTTGG |
| YY1 | ACCTGGCATTGACCTCTCAG | TTCTCATGGCCGAGTTATCCC |
| ACTB | CATCCTCACCCTGAAGTACCC | TAGAAGGTGTGGTGCCAGATT |
| TRPM1 | CAAAGATACATTCCCGTTTGC | GCTGAAAGAGCCTGAGCTGT |
| TYR | TACGGCGTAATCCTGGAAACC | CCGCTATCCCAGTAAGTGGA |
| SLC45A2 | AGAAGGGCCTCCACTACCAT | GTGAGCACCAATGCAGAGAAG |
| DCT | CCCCTACAGGGCCATAGATT | CCAGTAGGGCAAAGCAAAAG |
| SILV | ATAGGTGCTTTGCTGGCTGT | TCTGGGCTTCTGTCCACTCT |
| MLANA | CACTCTTACACCACGGCTGA | AATACCAACAGCCGATGAGC |
| GPNMB | GACAGCATGGTCAGAGGACA | GAACATCGTCCCAATTTCTGG |
| KIT | GGATCACGGAAAAGGCAGAA | GGCAGGATCTCTAACAAACACATAAA |
| CAPN3(total) | CAGATGGCTCTGGAAAGCTC | GCTGGTTGTTGAGGTGGAAT |
| CAPN3(isof) | CCAAAACATCCATCCTGAGC | GATTTGCCAACTCATTCCTTGA |
| CAPN3(isoa) | AAGTTCCCCATCCAGTTCGT | TCCCCTAGCTCTCCTTGACA |
| CAPN3(isod) | TGGGATGGAGGAATCACTTC | GAAGTCCTTCTGCAGGTGCT |
| CAPN3(isoe) | AATCCTGACCCTGAGCCAGT | ATCCCCACACACCTACCAC |
| COX2 | TCAGACAGCAAAGCCTACCC | GTTTTGACATGGGTGGGAAC |
| ICAM1 | CTGTTCCCAGGACCTGGCAAT | AGGCAGGAGCAACTCCTTTTTA |
| CXCL10 | GCCAATTTTGTCCACGTGTTG | GCTCCCCTCTGGTTTTAAGG |

ChIP-qPCR

| Gene | Forward | Reverse |
| --- | --- | --- |
| TRPM1 | AAAGCTCATGGAAAGCTGGAA | GCATCCACAGTCACCTGAAA |
| MLANA | GGATAGAGCACTGGGACTGG | CTGACGGGGTCGTCTGTAAT |
| GPNMB | GCAGTGCCGCTTAATACCAT | TGTTCTTCTGGCATCTGTGG |
| SLC45A2 (-1268~-1141) | AAGCCGTGTGTGATGTGTAAT | ACATGGGGCTTTTCTCGTTA |
| (-2408~-2554) | AGAAGGGCCTCCACTACCAT | ATCAGCTGACCCGTTCATTC |
| TYR(98-246) | TTGTACTGCCTGCTGTGGAG | CAGGAACCTCTGCCTGAAAG |
| (-1841~-1749) | CTTCCTGATTCCAGCCAGAG | ACAGAACAGGCTTTGCAGGT |
| DCT | CCCCTAATGAAGGCTTCTCC | TCTCCCTCCCTCCTTAGCTC |
| SILV(379~602) | CCAGAGCCCTTTCATGTGAT | CTCCAAAGGGGTGAAATGTG |
| (-5144~-5054) | AAGGGTGGTGGAGGCTAACT | CTCTGGGAATACAGGGGTGA |
| CAPN3(isoe) | TGTGTTGGGCTCTGTGTGTT | TGACTGTCACTGCCCAGAAG |
| KIT | ATAGATCCCGCTCCCATTTT | CAGTCACTTGGGCTTCCAAT |
