# Supplementary material for: YY1 Regulates Melanocyte Development and Function by Cooperating with MITF
Source: PLoS Genet. 2012 May 3;8(5):e1002688. doi: 10.1371/journal.pgen.1002688 (PMC3342948; doi:10.1371/journal.pgen.1002688)
Supplement: Text S1 — Supplemental methods. (DOC) [file pgen.1002688.s011.doc]

**Text S1**

Normalization of Paired ChIP-seq Data

NGS experiments may produce vastly different numbers of total reads for paired data, e.g. YY1 ChIP-seq vs. Input. Suppose there are *M* reads for IP and *N* reads for Input. Then, many researchers currently scale the Input counts per genomic window by *M*/*N*, in order to equalize the total number of reads between the two samples. This method, however, does not take into account that the IP data should accumulate a significantly greater number of reads in regions targeted by the antibody relative to the background; as a result, this scaling approach will artificially inflate the background noise captured by Input. A better alternative approach is to find a scaling factor that equalizes just the background between IP and Input channels without considering the IP peak regions.

In order to separate the antibody-targeted loci from background noise, we will apply the theory of order statistics. Our motivation comes from the following observation for normally distributed random variables: let *Y_1_*, *Y_2_*, ... , *Y_N_* be independent identically distributed normal random variables with mean μ and variance σ^2^, and let *Y*_(1)_, *Y*_(2)_, ... , *Y*(*_N_*) be their order statistics, i.e. the rearrangements of *Y_i_* such that *Y*_(1)_ ≤ *Y*_(2)_ ≤ ... ≤ *Y*(*_N_*). If we partition the reference genome into *N* equal-sized bins, then for sufficiently large μ, we can think of *Y_i_* as counting reads in the *i*-th bin. Define the partial mean $S_{n/N}$ as

$S_{n/N}=\sum_{k=1}^{n} Y_{(k)}/n$.

Because the order statistics are ranked in an increasing order, it can be seen that the partial mean is an increasing function of *n*. In fact, in the limit of large sample size *N*, the partial mean is almost a linear function of *n* with a positive slope. More precisely, for large *N*,

$S_{\alpha}\to\mu-\frac{\sigma}{\alpha}f(Y_{\alpha})$,

where *f* is the probability density of *Y_i_* and α=*n/N* [1]. Expanding this asymptotic form around α=1/2, one can show that the partial sum satisfies

$S_{\alpha}\sim^{N\to\infty} \mu-\frac{\sigma}{\alpha}\frac{(1-\pi(\alpha-1/2)^{2})}{\sqrt{2\pi}}$,

which is **almost linear in α** and can be fitted with linear regression with R^2^ > 0.99.

Similarly, consider a set of bivariate normal random variables *Z_i_* =(*Y_i_*,*X_i_*,), where *Y_i_* are defined as above and *X_1_*,*X_2_*,...,*X_N_* are independent identically distributed normal random variables that are uncorrelated with *Y_i_*. We can think of *Y_i_* as binned immunoprecipitated DNA counts and *X_i_* as binned Input DNA counts. Then, we define the *i*-th order statistic *Z*(*_i_*) to be the pair (*Y_k_*,*X_k_*), such that *Y_k_* corresponds to the *i*-th order statistic *Y*(*_i_*); i.e., the order statistics are obtained by sorting *Z_i_* with respect to the first entry. Because we have assumed that *X* and *Y* are uncorrelated, $\sum_{k=1}^{n} X_{(k)}/n$ is an unbiased estimate of the expectation $E[X]$, and for sufficiently large *n*, the ratio $R_{n/N}=\sum_{k=1}^{n} Y_{(k)}/\sum_{k=1}^{n} X_{(k)}$ of partial sums thus approaches $\sum_{k=1}^{n} Y_{(k)}/nE[X]$, which is proportional to the partial mean $S_{n/N}=\sum_{k=1}^{n} Y_{(k)}/n$. Consequently, for large *N*, the above analysis shows that $R_{n/N}$ can be **approximated by a linear function** of α=*n/N*.

In a more realistic situation, the distribution of the IP channel data *Y_i_* can be modeled as a mixture of two Poisson distributions, e.g. one component representing the basal level of background noise and the second component representing the enrichment of actual immunoprecipitated DNA. For sufficiently large mean, Poisson distributions approach normal distributions, and the above analysis still holds; but, the ratio $R_{\alpha}$ in this case begins to diverge from linearity at α roughly equal to the mixing probability. By computing this critical value of α, we can thus approximate the proportion of background noise in the IP channel data *Y_1_*, *Y_2_*, ... , *Y_N_*. The corresponding ratio $R_{\alpha}$ also provides the optimal scaling factor for normalizing the Input channel *X* in order to equalize the read counts in background regions that are not directly targeted by the antibody. This method can be applied to general paired ChIP-seq data, e.g. YY1 ChIP-seq vs. Input.

Detection of Peaks and Differentially Enriched ChIP-seq Regions

We have assessed the statistical significance of the differences between paired normalized ChIP-seq data as follows: let n_1_(x) and n_2_(x) be the normalized tag counts in a window centered at genomic location x in Sample 1 and Sample 2, respectively. Then, assuming the null hypothesis that n_1_(x) and n_2_(x) are independent and locally Poisson with common mean λ, the difference *Y*=n_1_(x)-n_2_(x) in tag counts follows the Skellam distribution

$$f(Y=n;\lambda)=e^{-2\lambda}I_{|n|}(2\lambda)$$

where the maximum likelihood estimate (MLE) of the mean is λ=(n_1_(x)+n_2_(x))/2. Because we have only two samples, the MLE may underestimate the true mean and increase the false positive rate. In our analysis, we have estimated the 95% confidence interval and used the more conservative value λ=0.5 qpois(0.975, n_1_(x)+n_2_(x)), where qpois is the quantile function in R for the Poisson random variable n_1_(x)+n_2_(x). For n > 0, the right-tail p-value is


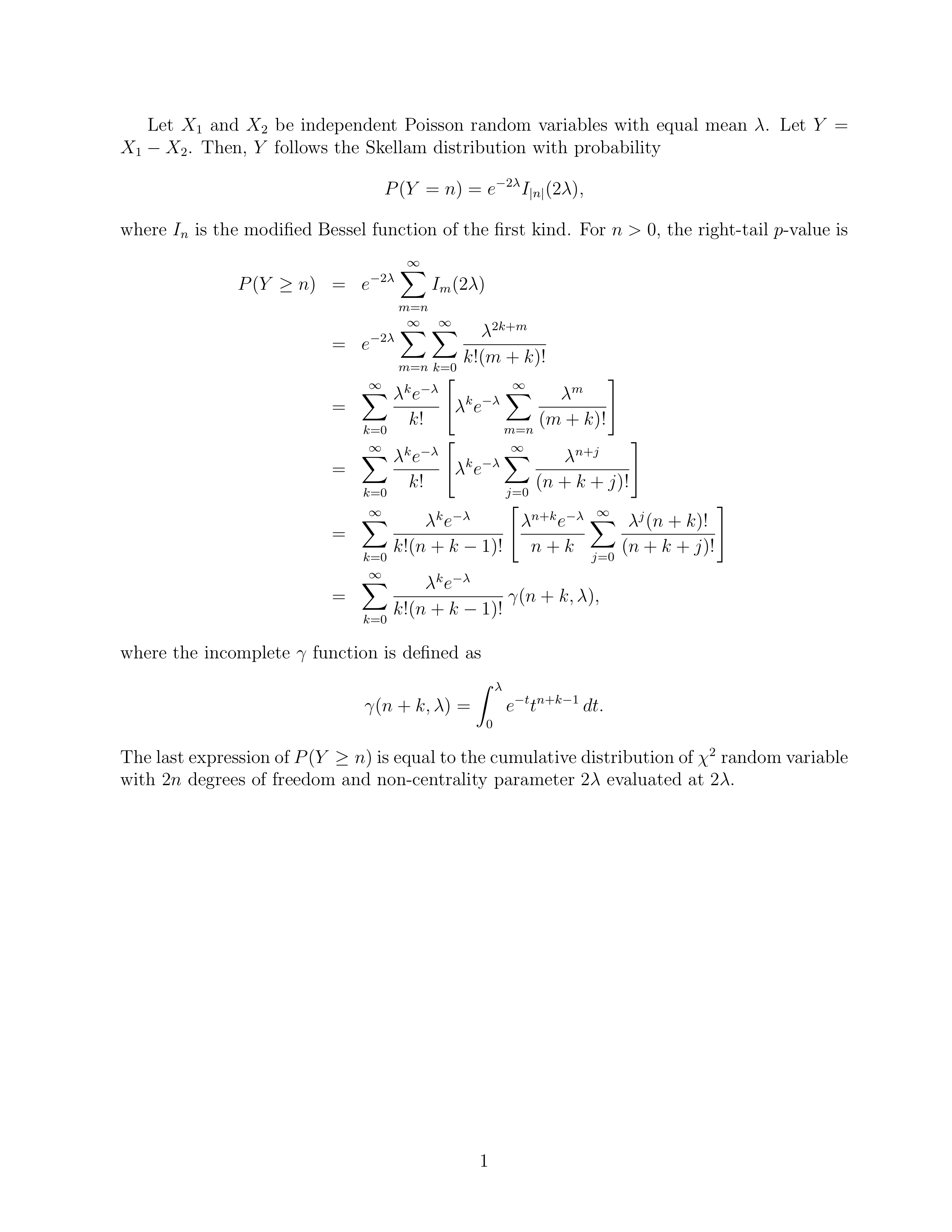


where the incomplete γ function is defined as


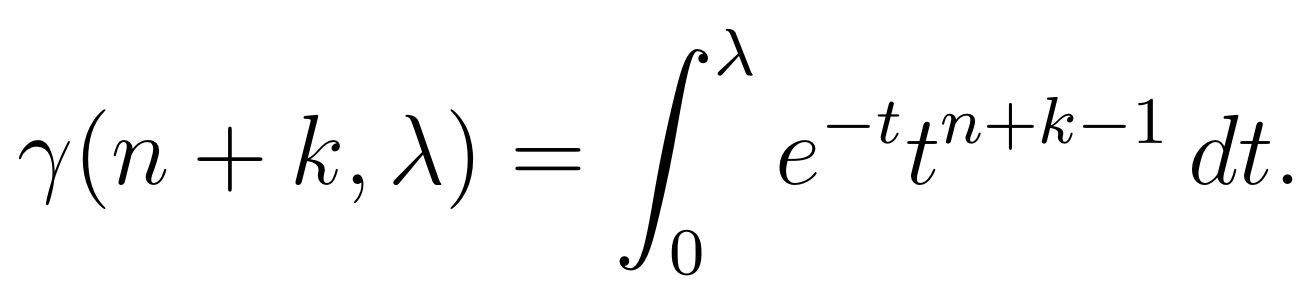


The final expression of the p-value is equal to the cumulative distribution of χ^2^ random variable with 2n degrees of freedom and non-centrality parameter 2λ evaluated at 2λ.

This novel method of detecting differential enrichment is more rubust than other approaches using Poisson *p*-values, which do not take into account the over-dispersion of count data and are thus more prone to false positives.

**References**

1. P.M. Burrows, *Expected Selection Differentials for Directional Selection.* Biometrics, 1972. **28**(4): 1091-1100.
